# Global mRNA selection mechanisms for translation initiation

**DOI:** 10.1186/s13059-014-0559-z

**Published:** 2015-01-05

**Authors:** Joseph Costello, Lydia M Castelli, William Rowe, Christopher J Kershaw, David Talavera, Sarah S Mohammad-Qureshi, Paul F G Sims, Christopher M Grant, Graham D Pavitt, Simon J Hubbard, Mark P Ashe

**Affiliations:** Faculty of Life Sciences, The University of Manchester, Michael Smith Building, Oxford Road, Manchester, M13 9PT UK; Faculty of Life Sciences, Manchester Institute of Biotechnology (MIB), University of Manchester, Manchester, M1 7DN UK; Current address: Biosciences, College of Life and Environmental Sciences, Geoffrey Pope Building, University of Exeter, Stocker Road, Exeter, EX4 4QD UK

## Abstract

**Background:**

The selection and regulation of individual mRNAs for translation initiation from a competing pool of mRNA are poorly understood processes. The closed loop complex, comprising eIF4E, eIF4G and PABP, and its regulation by 4E-BPs are perceived to be key players. Using RIP-seq, we aimed to evaluate the role in gene regulation of the closed loop complex and 4E-BP regulation across the entire yeast transcriptome.

**Results:**

We find that there are distinct populations of mRNAs with coherent properties: one mRNA pool contains many ribosomal protein mRNAs and is enriched specifically with all of the closed loop translation initiation components. This class likely represents mRNAs that rely heavily on the closed loop complex for protein synthesis. Other heavily translated mRNAs are apparently under-represented with most closed loop components except Pab1p. Combined with data showing a close correlation between Pab1p interaction and levels of translation, these data suggest that Pab1p is important for the translation of these mRNAs in a closed loop independent manner. We also identify a translational regulatory mechanism for the 4E-BPs; these appear to self-regulate by inhibiting translation initiation of their own mRNAs.

**Conclusions:**

Overall, we show that mRNA selection for translation initiation is not as uniformly regimented as previously anticipated. Components of the closed loop complex are highly relevant for many mRNAs, but some heavily translated mRNAs interact poorly with this machinery. Therefore, alternative, possibly Pab1p-dependent mechanisms likely exist to load ribosomes effectively onto mRNAs. Finally, these studies identify and characterize a complex self-regulatory circuit for the yeast 4E-BPs.

**Electronic supplementary material:**

The online version of this article (doi:10.1186/s13059-014-0559-z) contains supplementary material, which is available to authorized users.

## Background

In eukaryotic cells, the central hypothesis of molecular biology relies upon the transit of mRNA from the site of transcription and RNA processing in the nucleus through the nuclear pore to the translation machinery in the cytoplasm. The identification and selection of mRNAs in the cytoplasm for translation is widely acknowledged as fundamental to the regulation of gene expression [1–3]. This process relies heavily upon key modifications to mRNAs that are recognized by specific translation initiation complexes.

The vast majority of RNA polymerase II transcripts are processed at their 5’ end via the addition of a 7-methyl guanosine cap through a 5’-5’ triphosphate linkage, and at the 3’ end by addition of a polyadenylate (poly(A)) tail [4]. These mRNA modifications serve a number of functions, including increasing the translatability and the stability of the mRNA [5].

The 5’ cap structure is specifically recognized by the eukaryotic translation initiation factor (eIF)4E, a cup-shaped protein with a cap-binding pocket on its concave surface and a dorsal surface that is involved in protein-protein interactions [6–8]. Therefore, as part of the ‘typical’ cap-dependent translation initiation process, eIF4E binds to the mRNA cap in association with the eIF4G protein, as part of the eIF4F complex [9]. In contrast, eIF4E can exist in a translation repression complex bound to eIF4E-binding proteins (4E-BPs) [10]. The budding yeast *Saccharomyces cerevisiae* has two 4E-BPs - Caf20p and Eap1p - with roles in translational repression, although the precise conditions or pathways that elicit this repression are yet to be understood [11]. Current models for 4E-BP-mediated repression rely upon competition with eIF4G for interaction at an overlapping site on eIF4E [9].

eIF4G is a large factor which is thought to play a scaffolding role, coordinating interactions between translation initiation factors [12] such that, in the steady state, eIF4G exists in the eIF4F complex with eIF4E. Most likely as part of this eIF4F complex, eIF4G provides the crucial link to various translation initiation factors associated with the small ribosomal subunit, such as eIF3, eIF5 and eIF1A [13,14]. These interactions are thought to represent a critical part of the translation initiation process, as they facilitate the recruitment of the 40S ribosomal subunit with the initiator methionyl tRNA to the 5’ end of the mRNA, hence conveniently explaining the observation that initiation predominates at the first START codon from the 5’ end of an mRNA sequence [15]. Yeast and mammals have two eIF4G isoforms (eIF4G1/2 in yeast, eIF4GI/II in mammals). Yeast eIF4G1 and eIF4G2 are encoded by the *TIF4631* and *TIF4632* genes, respectively, and share 51% sequence identity [16]. Even though both genes complement the lethality of a double deletion mutant, early deletion experiments suggested some functional differences, as the *TIF4631Δ* strains are slow growing whereas *TIF4632Δ* strains grow as wild type [16]. More recent data suggest that any growth differences on rich medium relate to expression levels of the remaining eIF4G in the single mutant strains and that when the expression effects are genetically accounted for, there is no difference between strains bearing just a single eIF4G isoform [17]. Such experiments argue strongly that the eIF4G isoforms are functionally equivalent, although it is entirely possible that the situation may vary under different growth conditions.

Although the mRNA cap and the translation initiation factors bound to it are important in mRNA recognition, early experiments revealed that the 3’ poly(A) tail and the poly(A) binding protein (PABP generally, Pab1p in yeast) also play a role in eukaryotic translation initiation [12,18]. For instance, a range of experiments, including translation from *in vitro* extracts, microinjection studies and electroporation experiments, have shown that the presence of a poly(A) tail on a reporter mRNA increases the efficiency of protein production (reviewed in [18,19]). Furthermore, mutations in the *PAB1* gene in yeast impact on both translation and growth, and the lethality of a *pab1* null mutant is suppressed by factors involved in biogenesis of the large ribosomal subunit [20,21]. Over the years, a variety of mechanisms have been proposed to explain the impact of the poly(A) tail and PABP on translation. These include a role in large ribosomal subunit joining [22], a role in translation termination via eRF3 [23], and the closed loop model, where PABP and the poly(A) tail play a role in the recruitment of the small ribosomal subunit [5].

**Figure 1:**
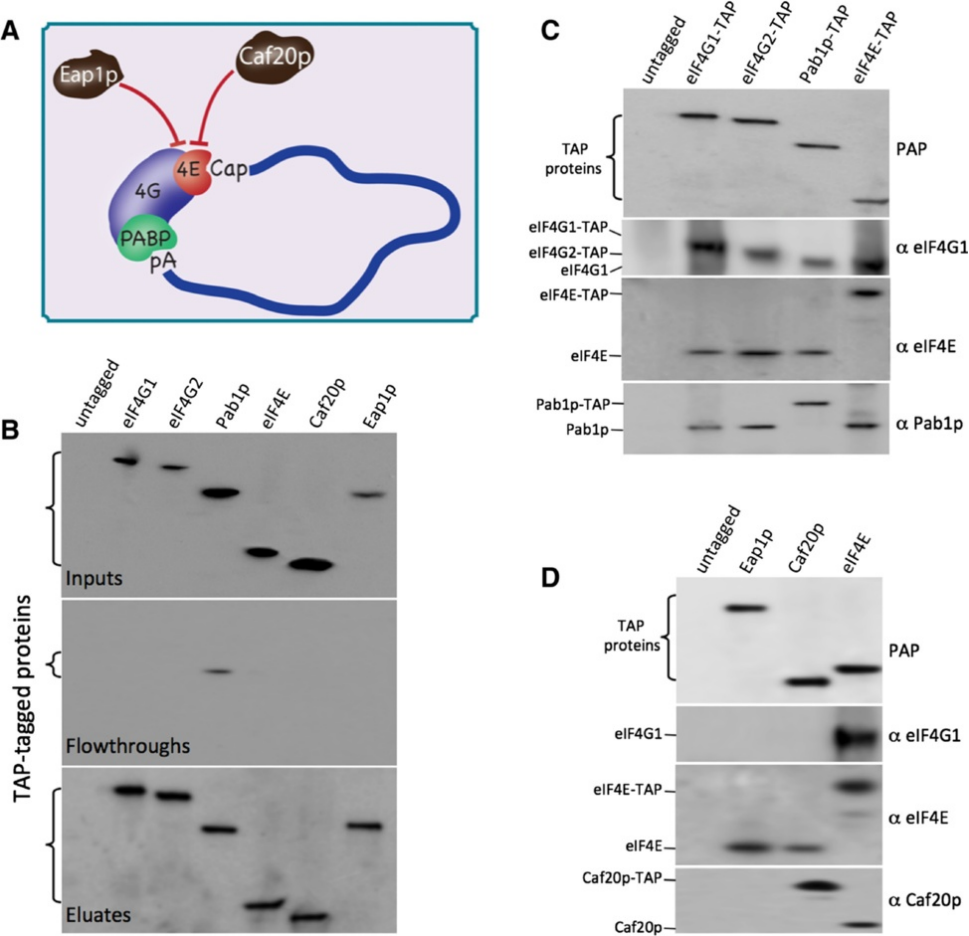
**The closed loop complex and 4E-BP repression complexes are maintained during purification. (A)** A diagram depicting the closed loop complex with eIF4E (4E), eIF4G (4G) and PABP bound to the mRNA; the 4E-BPs Caf20p and Eap1p are also represented competing with the eIF4E-eIF4G interaction. **(B)** Western blots probed with a protein A peroxidase (PAP) conjugate which detects the TAP-tagged proteins labeled above the blots. Input, flowthrough and eluates are presented on the top, middle and bottom blots respectively. The vast majority of TAP-tagged proteins purified appear in the eluates. **(C)** Western blots probed with PAP or the antibodies depicted on the right, which detect the components of the closed loop complex (either TAP-tagged or not) depicted on the left. Samples are eluates from TAP-affinity chromatography using strains bearing the TAP-tagged protein depicted above each lane. **(D)** As for (C), except the components of the 4E-BP complexes were assessed by TAP affinity chromatography.

Of these mechanisms, the closed loop model (Figure 1A), where a series of protein-RNA and protein-protein interactions bridge a molecular connection between the two ends of the mRNA, has received by far the most attention. The realization that both the 5’ cap and the 3’ poly(A) tail contributed to the translational efficiency of specific reporter mRNAs led to the first suggestions of a closed loop [24,25]. A critical development supporting such a model was that in electroporation studies and various *in vitro* translation systems, the presence of both a 5’ cap and a 3’ poly(A) tail on a mRNA resulted in a synergistic increase in translation initiation relative to that observed for mRNAs with only a single modification [26–28]. With increased biochemical understanding of the protein components and interactions involved in mRNA recognition came refinements to the model: where eIF4E interacts with the mRNA cap, PABP interacts with the poly(A) tail and eIF4G bridges the two ends of the mRNA leading to the formation of a closed loop [18]. Such a closed loop was observed with atomic force microscopy using purified components [29], and Pab1p has been shown to enhance the interaction of the eIF4G-eIF4E complex with the mRNA [30], with more recent data pointing to a dynamic interaction model where RNA structural alterations also impact upon the efficiency and lifespan of the interactions [31]. Further support for the closed loop model comes from yeast genetics. Mutations that affect interactions between the closed loop components inhibit translation initiation and prevent the cap-poly(A) synergy observed in translation extracts [32,33]. In addition, mutations affecting the eIF4E-eIF4G interaction are synthetic lethal in combination with mutations impacting upon the eIF4G-Pab1p and eIF4G-RNA interactions [33,34].

A variety of potential advantages to a closed circle of mRNA have been suggested [12,18]. For instance, it has been speculated that eukaryotic cells have evolved such a mechanism as a means to restrict efficient translation to intact mRNAs. Another possibility is that such an arrangement would facilitate the recycling of ribosomes on mRNAs. In this case, it might be anticipated that the reliance on the 5’ and 3’ ends would be more prevalent for mRNAs encoding highly translated proteins. Finally, the possible advantages of the closed loop complex in terms of mRNA stabilization have been considered. These have been further highlighted in studies into the mechanism of microRNA-mediated translational repression, where it has been postulated that microRNAs act to inhibit translation initiation via the GW182-dependent dissociation of PABP, which exposes an mRNA to the degradation machinery [35].

More recent data have challenged the requirement for an mRNA closed loop for translation initiation. For instance, evidence has been put forward suggesting that eIF4G is not required for all translation initiation events [36,37]. Indeed, in reconstituted systems, eIF4F is not necessary for recruitment of unstructured model mRNAs to the translation machinery [38,39]. Therefore, it is currently unclear whether the closed loop complex forms on all mRNAs to enhance their translation or is more specific.

To address which mRNAs interact with the closed loop and how these mRNAs are translationally regulated, we have taken a comprehensive RIP-seq strategy in the yeast *S. cerevisiae* to assess the mRNA binding profiles of the components involved: eIF4E, eIF4G1, eIF4G2, Pab1p, Caf20p and Eap1p. We find that eIF4E and eIF4G share almost identical mRNA binding profiles, supporting the idea that these proteins predominantly interact with mRNA in the form of the eIF4F complex. We also find that the profiles observed for eIF4G1 relative to eIF4G2 are almost identical, supporting the suggestion the two eIF4G genes functionally overlap in yeast [17]. Intriguingly, we observe two distinct classes of highly expressed mRNAs: those that are enriched with components of the closed loop complex and those that are under-represented with all of the closed components barring Pab1p. Intriguingly, Pab1p enrichment correlates better with ribosome density profiling analyses and global assessments of poly(A) tail length, suggesting that Pab1p interaction may act to support ribosome recruitment to stable mRNAs. The correlation with active translation for Pab1p extends further, as we observe an inverse correlation between the Pab1p mRNA binding profile and that of the yeast 4E-BPs, Caf20p and Eap1p. Finally, we identify the potential for a feedback control mechanism of the yeast 4E-BPs, where Caf20p exhibits enhanced binding to its own mRNA and that of Eap1p.

## Results and discussion

### Analysis of immunoprecipitated closed loop and 4E-BP complexes

The closed loop model represents a widely communicated explanation for the selection of mRNA for translation initiation [12,18,19]. We have evaluated the global mRNA binding profile of the components of the closed loop complex in *S. cerevisiae* (Figure 1A). We used strains bearing genomically integrated carboxy-terminal tandem affinity purification (TAP) tags on each of the endogenous genes encoding the components of the closed loop complex, and grew them under standard exponential growth conditions in batch culture. To provide a systematic analysis of the mRNA selection process and the regulatory mechanisms that may be involved, we also investigated the mRNA binding profile for each of the yeast eIF4E binding proteins (4E-BPs), Caf20p and Eap1p, which are considered repressors of translation (Figure 1A) [40].

In setting up the experimental system for immunopurification (IP) of mRNAs associated with each of these components, special attention was paid to a number of factors. First, the impact of TAP-tagging on the levels of each factor was assessed. As a result, we observed that in the *CDC33-TAP* strain, eIF4E-TAP protein is overexpressed, so we reconstructed this strain to remove the selectable marker and restore levels of eIF4E to wild type (data not shown). Second, all of the TAP-tagged strains used in this study were assessed in terms of both growth (data not shown) and global mRNA translation via polysome profiling (Additional file 1A). The strains carry the TAP-tagged allele as the only copy of these essential translation initiation factor genes; hence, the fact that the growth and polysome profiles are indistinguishable from the parent strain is suggestive that the tagged proteins are fully functional. Third, we developed a magnetic bead protocol for the rapid IP of proteins of interest such that our IP is complete within 20 minutes to minimise the impact on ribonucleoprotein complexes. Special care was also taken to optimise purifications so that the majority of each tagged protein was purified and hence depleted from the extract (compare inputs, flowthroughs and eluates in Figure 1B). In fact, the only IP where any TAP-tagged protein was detectable in the flowthrough was in the Pab1p sample. As a result, even though Pab1p is one of the most abundant RNA binding proteins in the cell [38], we still immunopurify over 80% of this protein from whole cell extracts (Figure 1B).

In order to ensure that the IPs contain proteins that are consistent with the formation of both the closed loop complex and 4E-BP repression complexes, immunopurified samples were probed with various antibodies on western blots (Figure 1C,D). On these blots, migration of each TAP-tagged protein is retarded relative to the untagged protein (for example, Pab1p-TAP, eIF4E-TAP and Caf20p-TAP in Figure 1C,D). For eIF4G1-TAP and eIF4G2-TAP, protein A in the TAP epitope is detected by the secondary antibody; hence, an eIF4G2-TAP protein band is observed even though an eIF4G1 specific primary antibody was used (Figure 1C). However, to summarise this analysis, all of the relevant components of the closed loop complex, eIF4E, eIF4G1 and Pab1p, were present in the appropriate IPs of eIF4E, eIF4G1, eIF4G2 and Pab1p (Figure 1C). Equally, in terms of the 4E-BP repression complexes, both Eap1p and Caf20p IPs contain eIF4E but not eIF4G1 (Figure 1D), consistent with the generally accepted competitive model for 4E-BP-mediated repression depicted in Figure 1A [41]. Finally the capacity of the TAP-tagged proteins to interact appropriately with the mRNA cap was assessed using Cap affinity chromatography (Additional file 1B). Each of the TAP-tagged components was isolated on the resin in a similar manner to the relevant untagged protein, suggesting that the TAP tags in the strains are not unduly influencing the capacity of the tagged proteins to interact with RNA or proteins bound to RNA.

### Enriched mRNAs and the functional significance of their association

**Figure 2:**
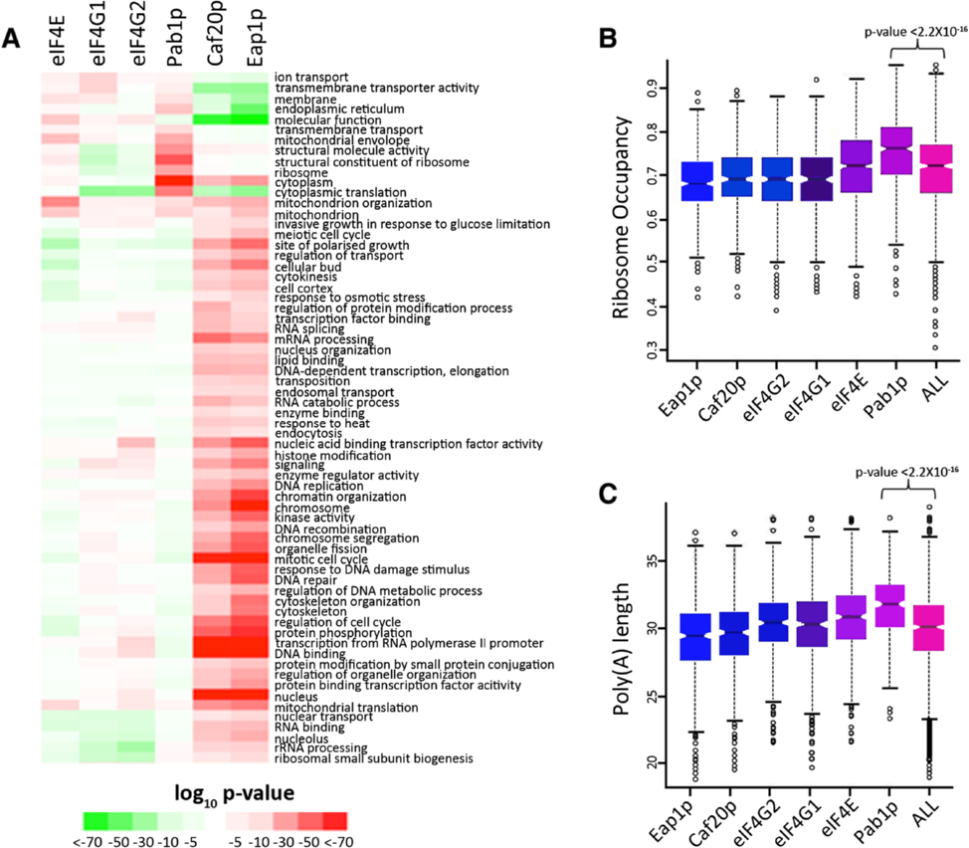
**Properties of transcripts preferentially associated with closed loop components and 4E-BPs. (A)** Gene Ontology terms that are significantly over-represented (red) or under-represented (green) for each of the transcript sets enriched in the affinity pulldown of the six proteins shown at the top. **(B,C)** Box and whisker plots detailing the variation in ribosome occupancy [42] and poly(A) tail length [43] for the transcripts enriched with the closed loop components and 4E-BPs. A Wilcoxon rank statistical test between transcripts sets revealed a high association for both the features with Pab1p-enriched transcripts. On these plots the coloured boxes depict the extent of the upper and lower quartiles with the notch representing the median. The dotted capped vertical lines depict the upper and lower extremes while the circles are outlying values.

The mRNAs that are associated with the IPs for eIF4E, eIF4G1, eIF4G2, Pab1p, Caf20p and Eap1p across three biological replicates were quantified by RNA-seq (see Materials and methods for details, and Additional file 2 for mapping statistics and counts). The data were processed to generate a ratio for each mRNA species relative to a total RNA sample from the same TAP-tagged strain. Libraries were sequenced to an average depth of 7.8 ± 5.8 million unique, mapped reads, and the data were used to establish lists of statistically (false discovery rate (FDR) <0.05) enriched transcripts in the immunoprecipitated sample using the GLM paired model of EdgeR (complete lists provided in Additional file 2). A functional enrichment analysis generated from the RIP-seq data is presented in Figure 2A, from which a number of trends are evident. Both of the eIF4E binding proteins, Caf20p and Eap1p, have IP-enriched transcripts whose protein products participate in a whole host of nuclear functions (for example, RNA processing and transcription), possibly revealing a connection between translational regulation and the regulation of upstream events in the gene expression pathway. In a previous study, we assessed the functional significance of Caf20p and Eap1p through the use of microarrays to measure any change in association of mRNAs across polysome gradients in wild-type versus *caf20Δ* and *eap1Δ* mutant strains [40]. This revealed that over a thousand transcripts were potentially regulated at the translational level by the yeast 4E-BPs. Here, we can assess how the mRNAs found to be associated with Caf20p and Eap1p through RIP-seq change in terms of polysomal association in the knockout strains. Transcripts that preferentially associate with Caf20p and Eap1p are shifted subtly, but significantly (using a range of FDR cutoffs), up into the polysome fractions in the *caf20Δ* strain relative to wild type (Additional file 3). For the *eap1Δ* mutant strain relative to the wild type similar trends were observed across the *eap1Δ* plots, although the scale of effect is significantly less pronounced than for the *caf20Δ* data (data not shown), potentially owing to its reduced abundance in the cell with respect to Caf20p [38]. Overall, this comparison of the RIP-seq data with previous microarray studies on mutants is consistent with the described role for the 4E-BPs as repressors of translation initiation.

To assess other correlations for the individual gene lists, they were compared with a host of different parameters, including poly(A) tail length [43] and ribosome occupancy [42] (Figure 2B,C). Surprisingly, the mRNAs associated with Caf20p and Eap1p have ribosome occupancies that are as high as mRNAs associated with either eIF4G1 or eIF4G2. There could be a number of reasons for this: the interaction with eIF4E might not be the only manner in which Caf20p and Eap1p can associate with mRNAs, or the yeast 4E-BPs may simply dampen the translation of highly translated mRNAs such that, on average, the 4E-BP-associated mRNAs can still have a high ribosome occupancy. Perhaps the most striking result from these comparisons is that the Pab1p-enriched transcripts are associated not only with longer poly(A) tails, as might be expected, but also with the high levels of ribosome occupancy (Figure 2B,C). The high level of correlation between poly(A) tail length and ribosome association has been observed before [43], but here we show that the level of ribosome occupancy for the Pab1p-enriched transcripts was significantly higher than for transcripts associated with other components of the closed loop complex (Figure 2C). These results highlight Pab1p as an important player for mRNAs where translation is particularly efficient and robust.

### The Pab1p RNA binding profile is different to that of the other closed loop components

**Figure 3:**
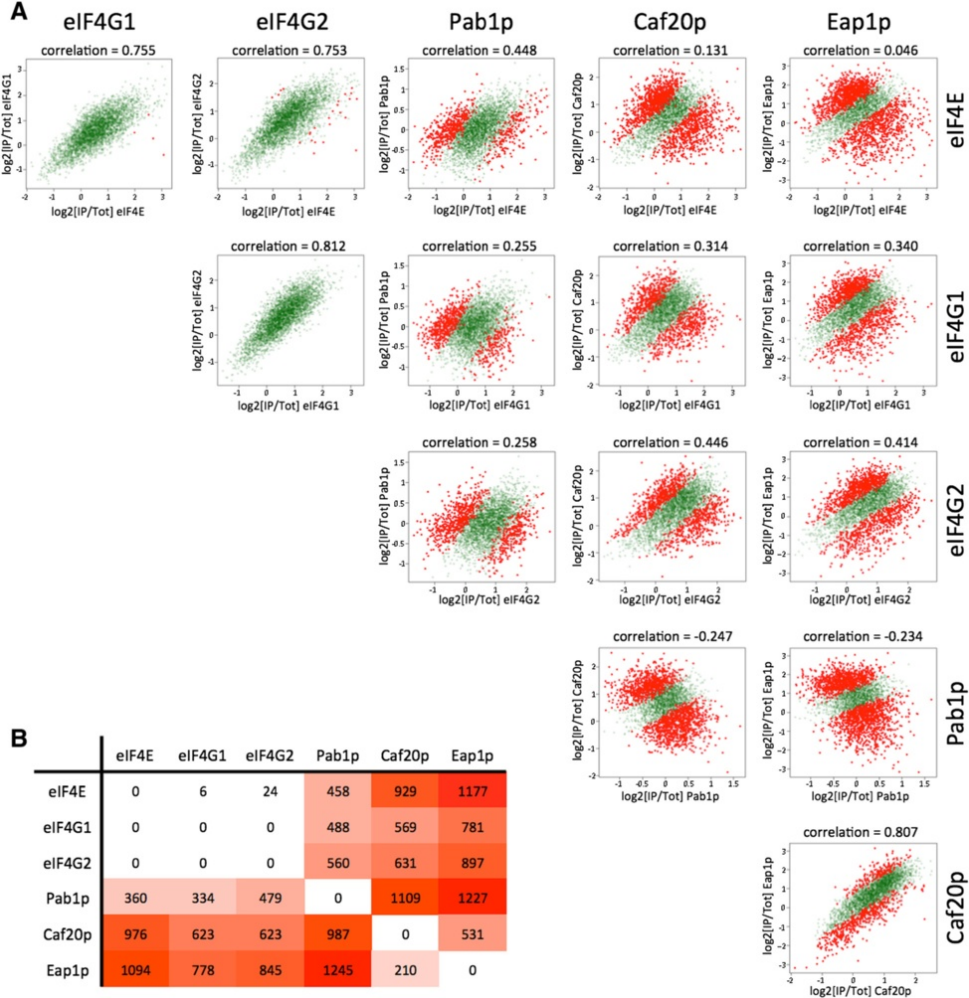
**Direct pairwise comparisons between RIP-seq experiments for each of the closed loop components and 4E-BPs. (A)** Scatterplots display change in the log_2_ median fold changes (IP/Total) for each of the six proteins compared with one another. Highlighted in red are those transcripts identified as being significantly different between the two experiments according to edgeR's interaction GLM model at a FDR <0.05. **(B)** Table depicting the total numbers of transcripts that vary significantly across the pairwise comparisons in **(A)**. The numbers represent transcripts that are over-represented in the IPs of proteins listed in the columns relative to proteins listed in each row.

In order to provide a quantitative assessment of the variation between different RIP-seq datasets in a pairwise fashion, we have used an interaction model derived from the Generalised Linear Model (GLM) function within the EdgeR software package (Bioconductor) [44,45]. More specifically, we compared the ratio of mRNA levels in the IP samples relative to the level in a total RNA sample (log_2_(IP/Total)) for each gene in each of the six immunoprecipitation experiments, and examined the pairwise correlations between them (Figure 3A). Here the full profile of mRNA enrichment values is presented, rather than those defined by a statistical cutoff. These data are presented as scatterplots cross-comparing the datasets, highlighting in red the transcripts found to be significantly different between the experiments according to the GLM. Strikingly, these plots emphasize the high correlation observed in the binding profiles of the three members of the eIF4F complex (Pearson correlations of 0.755, 0.753 and 0.812). Likewise, the two 4E-BP binding profiles, for Caf20p and Eap1p, also display a similar high correlation with each other. Notably, while Pab1p displays positive correlations with components of the eIF4F complex, it is the only factor assessed that displays a negative correlation with the profiles from the translational repressors Caf20p and Eap1p.

The numbers of transcripts at variance with the interaction model are shown in Figure 3B (detailed in Additional file 4). This shows the numbers of differentially enriched or under-represented transcripts for each pairwise comparison according to the GLM (the red data points), and supports the general trends observed in the scatterplots (Figure 3A). For example, no transcripts were identified as significantly different in their association with the two eIF4G isoforms, eIF4G1 and eIF4G2. This observation is consistent with recent data suggesting that these two isoforms are likely to be functionally redundant [17]. These results, combined with the data above, suggest that the eIF4F complex explains the vast majority of the interactions of eIF4E, eIF4G1 and eIF4G2 with mRNA. This is particularly intriguing given that the translational repressors Caf20p and Eap1p inhibit translation via interaction with eIF4E, yet their binding profile exhibits minimal correlation with that of eIF4E. Possible explanations for this are that the 4E-BPs may interact with mRNAs in ways that are independent of eIF4E or that the 4E-BP-eIF4E complex may be less stably associated with mRNA.

It is also apparent that if Pab1p were stoichiometric with the eIF4F complex on every mRNA, then the mRNA binding profile for Pab1p would be expected to be similar to that of eIF4E, eIF4G1 and eIF4G2. However, this is clearly not the case. It should be noted that Pab1p binds the 3’ end of the mRNA and the eIF4F components associate with the 5’ end. It is possible that this difference explains some of the variation that is apparent between these RIP-seq datasets. Alternatively, these data might highlight that the closed loop complex is only relevant for the translation of a subset of mRNAs. Intriguingly, the Pab1p profile inversely correlates with those of the translational repressors Caf20p and Eap1p (indeed, it rather than the eIF4G profiles is by far the strongest anti-correlation with 4E-BP profiles), and the Pab1p-enriched transcripts also have higher ribosome occupancy (Figure 2B), emphasizing a strong correlation between Pab1p association and active translation.

### Clustering of RIP-seq enrichment profiles reveals global trends in translational control via the closed loop complex and Pab1p

**Figure 4:**
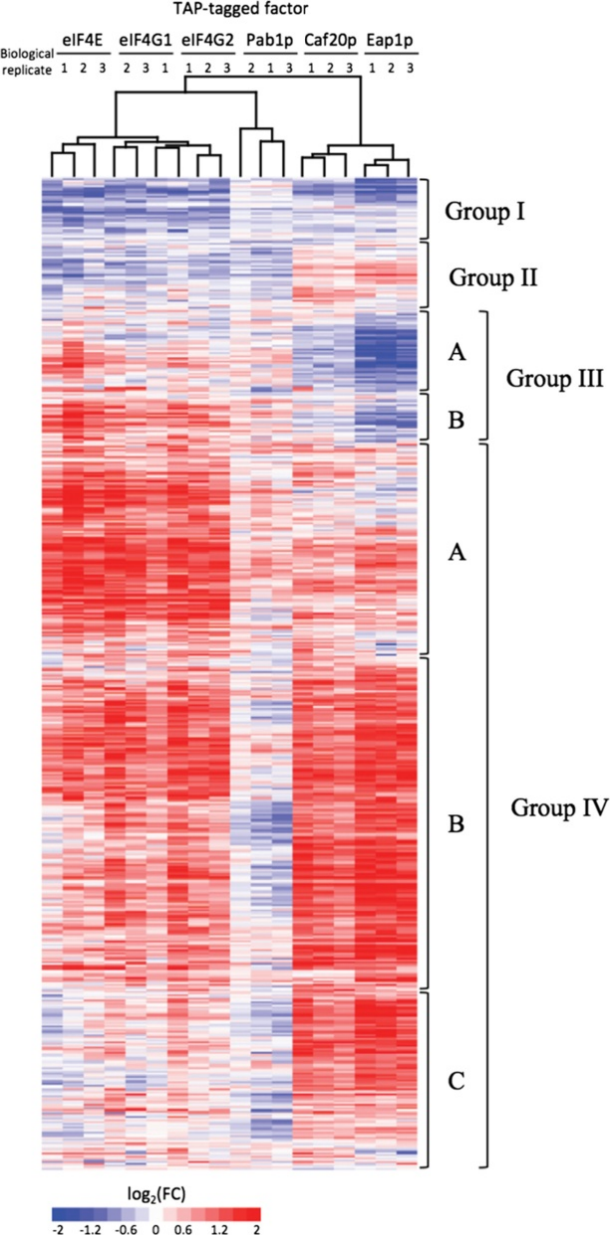
**Four major clusters are apparent across the RIP-seq datasets.** Heatmap derived from hierarchical clustering of the mRNA binding profiles (log_2_ fold changes IP/Total) of the six proteins in the study; red and blue represent over- and under-represented mRNAs, respectively. The analysis was restricted to 3,173 transcripts that were identified as over-represented or under-represented in any of the six datasets and hierarchical clustering was performed as described in the Materials and methods section. The heatmap presented contains 2,767 transcripts separated across 7 clusters that have been grouped into groups I to IV based upon similarity of the patterns of association.

While the pairwise comparison of the RIP-seq datasets has provided significant insights, comparison of the RNA binding profiles across all of the RIP-seq datasets can be visualised simultaneously using a hierarchical clustering method. The RIP-seq data were therefore expressed in the form of a heatmap, displaying the three strongly correlated biological replicates for each closed loop member as columns, and individual transcripts as rows (Figure 4). In order to ensure a minimal number of false positives, a more conservative statistical cutoff coupled with a count-based filter was used for the clustering analysis. More specifically, the heatmap is restricted to those transcripts displaying a significant (FDR <0.01) enrichment or under-representation according to EdgeR’s GLM model in at least one of the IPs, as well as to transcripts with greater than 20 reads in each of the pertinent total extract samples. In total, 3,173 of the annotated genes in the yeast genome satisfy these criteria. Hence, a substantial proportion of the yeast transcriptome shows little discernable, statistically significant enrichment or under-representation with respect to total mRNA, and the heatmap focuses on those that do.

Using standard hierarchical clustering (see Materials and methods for details) of the RIP-seq enrichment profiles, four broad groups (I to IV) can be defined which encompass 2,767 transcripts (Figure 4; Additional file 5). These four visually distinct groups exhibit intriguing patterns of association with the closed loop components and the translational repressors Caf20p and Eap1p, manifest largely as blocks of enrichment/under-representation with respect to eIF4E/4G1/4G2/Pab1p and Caf20p/Eap1p. Group I contains mRNAs that are mostly under-represented in IPs from all of the components tested with the exception of Pab1p. Group II contains mRNAs enriched in IPs of the translation repressors Caf20p and Eap1p. Group III consists of two clusters that contain mRNAs that are enriched in closed loop component IPs but under-represented in the Caf20p and Eap1p samples: a profile of association that might be expected for mRNAs where translation is highly active and initiated robustly via the closed loop complex. Finally, group IV is a large, broad group of mRNAs that are enriched across both the eIF4F and 4E-BP datasets, with three subclusters determined by level of enrichment with Pab1p or the eIF4F components; here there may be a complex competition between the interaction of closed loop components and the translation repressors. It is particularly intriguing that roughly half the mRNAs in group IV are under-enriched for Pab1p even though these same mRNAs are enriched with eIF4F. It seems plausible that, for these mRNAs, the poly(A) tail could interact with other RNA binding proteins such as Nab2p or Sgn1p, which have been suggested to compete with Pab1p previously [46]. Overall, these clusters highlight the possibility that translation initiation via the closed loop complex could be more important for some mRNAs than others. In particular, group III defines mRNAs that appear to interact preferentially with the closed loop complex, and group II mRNAs that interact preferentially with the 4E-BPs.

Before considering these global translational groups of mRNAs in more detail, it is interesting to note that, as previously found, using the GLM model and the scatterplots (Figure 3), there is a high correspondence between the eIF4E, eIF4G1 and eIF4G2 IP profiles whereas the pattern for Pab1p appears different (Figure 4). In addition, while the profiles for the yeast 4E-BPs, Caf20p and Eap1p, are very similar, they are different to the patterns observed for the other IPs. This is particularly evident in the clustering dendrogram presented over the columns in Figure 4. Indeed the eIF4G1 and eIF4G2 profiles are so similar as to be indistinguishable across the dendrogram. The fact that the eIF4F components eIF4E and eIF4G exhibit a similar mRNA interaction profile whereas that for Pab1p appears different again points towards a model where the full closed loop complex is only relevant for the translation of a subset of mRNAs. Furthermore, since in group II the 4E-BPs can be identified as interacting preferentially with transcripts where the translation factors are not enriched, it seems that the straightforward model of eIF4E-4E-BP interaction on mRNAs to repress translation may be an over-simplification.

**Figure 5:**
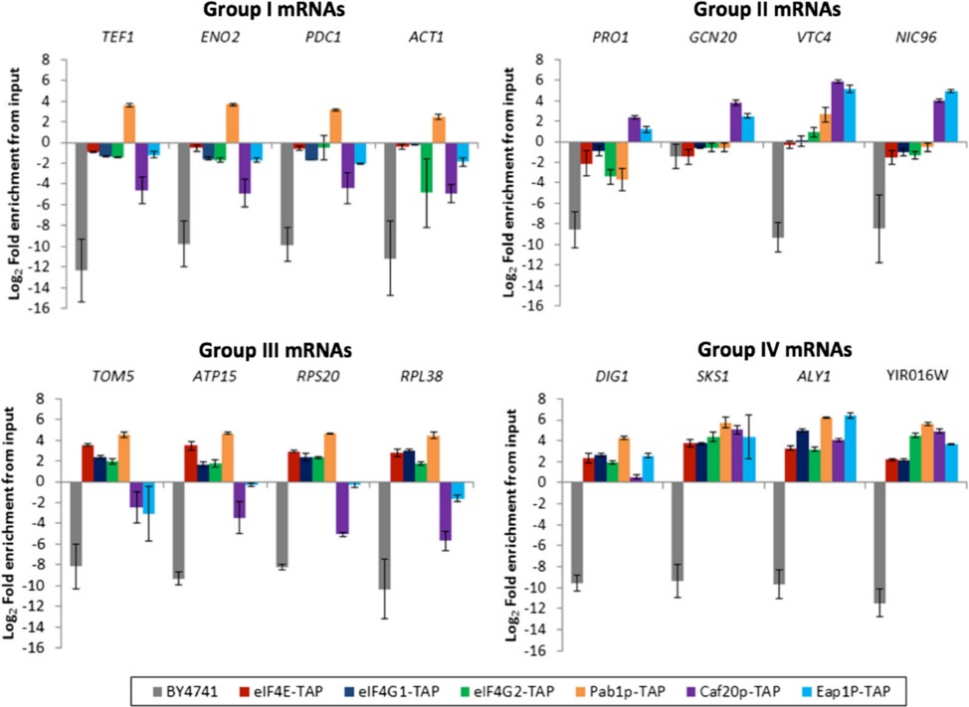
**Validation of transcript clusters by quantitative RT-PCR.** Figure shows four plots, one for each of the transcript clusters. The indicated mRNAs are quantified in the IP samples relative to total RNA for the untagged control and closed loop/4E-BP regulatory components. Error bars are ± standard error from three replicate experiments.

In order to provide an independent validation for the clusters identified in Figure 4 and the RIP-seq datasets as a whole, a series of quantitative reverse transcriptase PCR (qRT-PCR) analyses were conducted on RNA prepared from IPs of the TAP-tagged factors and compared with the levels of RNA in the input fractions (Figure 5). These data exhibit an excellent correlation with the RIP-seq analysis. Group III mRNAs are enriched with eIF4E, eIF4G1, eIF4G2 and Pab1p but not the 4E-BPs. Group II mRNAs are predominantly enriched with the 4E-BPs. Group IV mRNAs are enriched for most of the TAP-tagged components and group I mRNAs are under-enriched for all factors except Pab1p. This last observation is particularly striking and is suggestive that Pab1p plays a key role in the translation of these high abundance mRNAs. Pab1p has been suggested to enhance various stages in the translation process, including subunit joining during initiation [22] and translation termination/ribosome recycling [23]. Therefore, one possibility is that Pab1p acts to enhance these steps independently of cap-interacting proteins. Alternatively, Pab1p could be acting in a hitherto unidentified fashion.

Another aspect of the data that is highlighted by the qRT-PCR analysis is that despite the under-enrichment of transcripts with various closed loop components and 4EBPs relative to a total RNA sample, the mRNAs are still present in the IPs when compared with the level of mRNA obtained from an untagged strain (Figure 5; compare BY4741 with the TAP-tagged strains). This is also apparent from the RIP-seq data as measured by the RPKM values (reads per kilobase of transcript per million reads mapped) in the IP experiments (Additional file 2). That is to say, even those mRNAs that are under-enriched with eIF4F, Pab1p or the 4E-BPs are clearly still bound by these components, albeit at a significantly reduced level.

### Functional analysis of enriched mRNAs and closed-loop clusters

**Figure 6:**
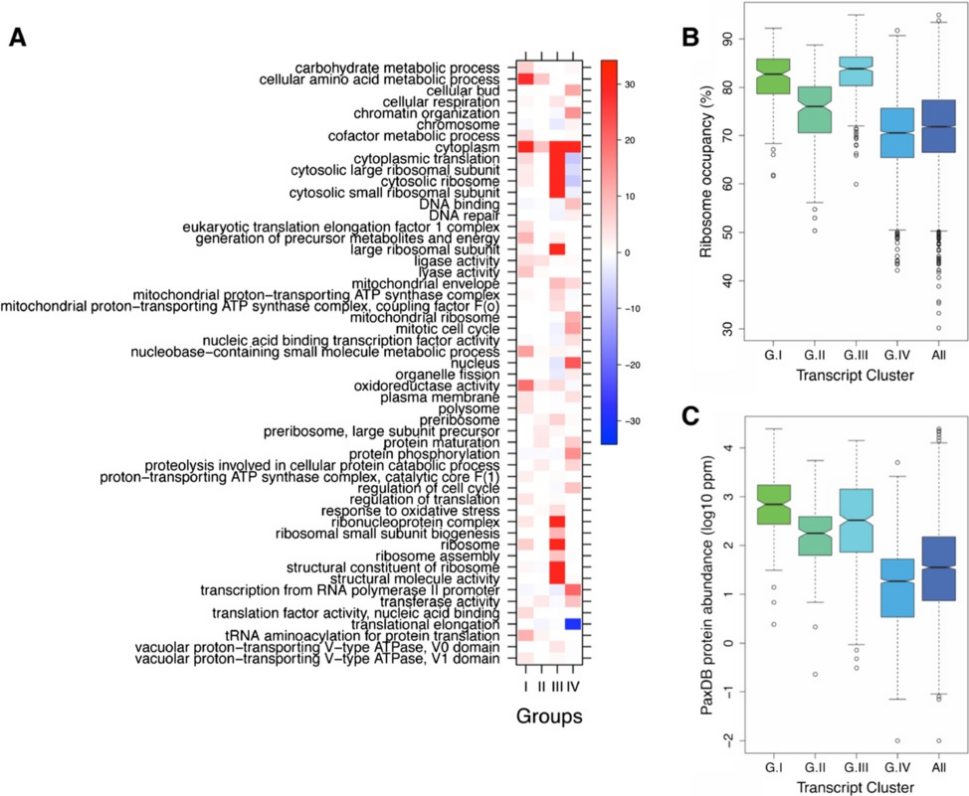
**Functional analysis of proteins encoded by the transcript clusters. (A)** Gene Ontology (GO) terms that are significantly over-represented (red) or under-represented (blue) for transcripts that are present within the four clusters defined in Figure 4. Only the GO terms that show significant differences (via a Fisher test comparing that cluster with the rest of the genome; FDR <0.01) for at least one cluster and also had differences between the clusters (Chi-square test; FDR <0.01) are depicted. The color scale represents the statistical significance of GO term enrichment or under-enrichment as measured by log_10_FDR. For convenience the GO term enrichment log_10_FDR values have been multiplied by -1. **(B,C)** Box and whisker plots, as in Figure 2, detailing the variation in ribosome occupancy [42] and protein abundance according to the PaxDb database [49] for the transcripts enriched with the closed loop components and 4E-BPs. A Wilcoxon rank test between pairwise transcripts sets revealed a significant difference (*P* < 1 × 10^-7^) in all cases for both plots; the only exception being between group I and group III for ribosomal occupancy, which was significant at *P* < 0.03.

To further decipher the functional role of the closed loop components in translation initiation in yeast, we examined the groups of mRNAs derived from the heatmap for trends and patterns in terms of gene function. Initially we focussed on group III, as this group exhibits the clearest pattern of association with closed loop complex components. We found that the group III mRNAs exhibit high ribosome occupancy and their protein products are, on average, highly abundant (Figure 6B,C). This is consistent with closed loop complex-dependent translation initiation acting as an efficient route for the production of highly abundant, stable proteins. A functional analysis of the mRNAs present within this group lends further weight to this idea, as it demonstrates a very substantial enrichment in mRNAs for ribosomal proteins (Figure 6A); 115 of the 395 genes in group III encode ribosomal proteins. The fact that the mRNAs for the ribosomal proteins are heavily enriched with the closed loop machinery provides an interesting parallel with the situation in mammalian cells where many of the mRNAs encoding ribosomal proteins display discrete regulatory patterns by virtue of a 5’ terminal oligopyrimidine (TOP) motif [47]. No such *cis*-acting sequences are obvious in either the yeast ribosomal protein mRNAs or across the group III mRNA set (data not shown); however, it seems likely that such elements must dictate the very high level of association that we observe with the closed loop complex components. The parallels between the group III mRNA properties and those of mammalian TOP mRNAs run deeper. For instance, TOP mRNAs are especially sensitive to regulation by mammalian target of rapamycin (mTOR), and recent evidence suggests this occurs via the 4E-BP1 translation repressor [48]. The specific regulation of the TOP mRNAs in this manner suggests that these mRNAs are highly sensitive to cap complex and possibly closed loop complex inhibition [48]. Therefore, the specific enrichment of ribosomal protein mRNAs with components of the yeast closed loop complex highlights the possibility that a parallel mechanism could exist in yeast.

Similar to group III, the mRNAs from group I have high ribosome occupancies and their protein products are highly abundant (Figure 6B,C). However, the pattern of association with the closed loop components is very different for group I relative to group III. Group I mRNAs are generally under-represented for eIF4E, eIF4G1, eIF4G2, Caf20p and Eap1p. Indeed, the IPs of Pab1p are the only ones where this group of mRNAs is not under-represented relative to total (Figures 4 and 5). An analysis of the functions of the protein products of the mRNAs within this group highlights amino acid and nucleotide metabolism, carbohydrate metabolism/energy generation, tRNA amino-acylation and translation (Figure 6A; Additional file 5). Strikingly, this group includes mRNAs encoding the glycolytic factors, such as *ENO2*, *TDH3* and *PGK1*, and translation factors, such as *TEF1*, *TEF2*, *EFB1*, *TIF1* and *TIF2*, which are amongst the most highly expressed and hence abundant proteins in the cell. The under-representation of the mRNAs in this group with eIF4F components combined with the high abundance of the protein products is suggestive that the closed loop complex is much less relevant for high efficiency translation initiation for this group of mRNAs than for the group III mRNAs. It is plausible, therefore, that such mRNAs require an alternative to the closed loop mechanism for their highly efficient translation initiation. One possibility is that Pab1p is somehow involved in such an alternative high efficiency mechanism, especially considering that group I mRNAs are under-represented with all of the closed loop protein components barring Pab1p. Interestingly, with regard to such a model, mRNAs that are statistically over-represented in Pab1p IPs exhibit, on average, longer poly(A) tails and higher than average ribosome occupancy (Figure 2B,C). Pab1p has been previously suggested to act at multiple steps in the translation process: internal initiation, 60S ribosomal subunit joining and translation termination. It is possible, therefore, that one of these activities explains the observation that the group I mRNAs appear heavily translated even though they interact less well with the eIF4F components. The observations for this cohort of mRNAs combined with the inverse correlation between Pab1p and the 4E-BPs in terms of their mRNA binding profiles adds to a picture where Pab1p interaction represents a key predictor of translation efficiency.

The group II mRNAs from the heatmap include mRNAs that are preferentially associated with the 4E-BPs, and hence should be translationally repressed. As above, even though these transcripts appear under-enriched for eIF4E, the mRNAs are clearly still present in the IP experiments but are under-enriched relative to other heavily bound transcripts. A functional analysis of the mRNAs from group II identifies strong enrichment for certain amino acid biosynthetic pathways, including those for the hydrophobic and basic amino acids (Figure 6A; Additional file 5), although it should be noted that many amino acid biosynthetic genes are also present in groups I and IV. However, intriguing connections have been identified between the Gcn pathway controlling amino acid biosynthesis and the yeast 4E-BPs [50]. Overall, the finding that specific mRNAs are enriched with the 4E-BPs is consistent with the assumption that such mRNAs are not critical in unlimited, exponential growth; hence, translation is repressed by virtue of the 4EBP repressors. This analysis is also consistent with the hypothesis that the yeast 4E-BPs are not global regulators of translation initiation but instead function to regulate in a mRNA-specific manner [40,51,52].

Finally, the large group represented by group IV is characterized by strong interactions with both eIF4F and the repressive 4E-BPs. The proteins encoded by this group of mRNAs display a very broad range of functions; this group is enriched in functions linked to transcription, protein phosphorylation, and the cell cycle, and is under-enriched for functions linked to translation and the ribosome. This group contains 79 of the 127 protein kinase encoding mRNAs, whereas no other group contains any protein kinase mRNAs. Therefore, it appears that this group contains mRNAs for processes that are tightly regulated in the cell, including signalling and activation of pathways and responses to stimuli; our data suggest that some of this is manifest at the translational level. We suggest these processes are under finite control where a delicate balance exists in the level of an individual mRNA bound by the closed loop relative to the 4E-BPs. It may also be true that this group is poised such that derepression of translation via the relief of 4E-BP repression would represent a means of releasing a 'molecular handbrake'.

### Estimation of the stoichiometry of protein interactions with mRNA-bound eIF4E

**Figure 7:**
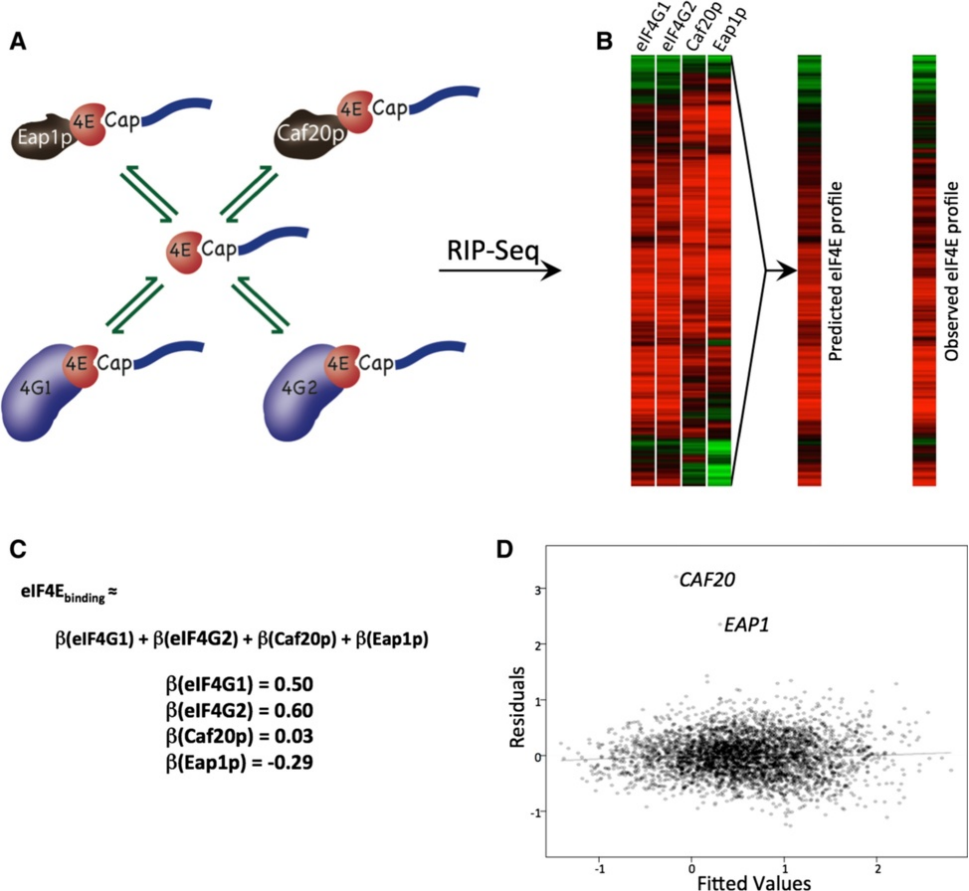
**A stoichiometric model for eIF4E binding highlights the** ***CAF20*** **and** ***EAP1*** **mRNAs as significant outliers. (A)** Diagram showing the theoretical basis for the stoichiometric model where the four proteins, eIF4G1, eIF4G2, Caf20p and Eap1p, all bind the cap binding protein eIF4E, which in turn binds mRNAs in a complex equilibria. This model presumes the eIF4E mRNA binding profile can be modeled via a linear combination of the other closed loop protein profiles. **(B)** Heatmaps of mRNA enrichment for each of the four eIF4E binding proteins, and a predicted mRNA enrichment heatmap for eIF4E based on a best fit of the model to the RIP-seq data, which in turn can be compared with the observed mRNA enrichment for eIF4E. An excellent correlation is observed between the predicted and observed heat map profiles, with R^2^ = 0.75. **(C)** Equation detailing the assertion that the binding profile for eIF4E is equal to the sum of the profiles observed for all of the eIF4E binding proteins, with the corresponding β coefficients representing the contribution of each individual profile to the overall model (all significant *P* < 0.002). **(D)** A plot showing the fitted model values and the corresponding residuals from the linear regression modeling. Notably, the *CAF20* and *EAP1* mRNAs are significant outliers from the model when plotting their residuals from the predicted eIF4E enrichment values.

The finding that a large group of mRNAs are overrepresented in immunoprecipitations of both eIF4G and the 4E-BPs (Figure 4, group IV mRNAs) highlights the potential for competitive interactions with eIF4E on the mRNAs between the various eIF4E binding proteins. Working from an assumption that the sum of the interactions of the four eIF4E binding proteins should approximate to the RNA binding profile for eIF4E as the gatekeeper for initiation (and hence translation; Figure 7A,B), we can express this mathematically via a simple linear combination of the four profiles (Figure 7C).

Here the β values are coefficients representing the nominal contributions of each eIF4E binding protein to eIF4E’s binding profile, taking the eIF4E profile as a general proxy for cap-dependent initiation. We can estimate the values of the β coefficients in the above equation using standard multiple-linear regression to build a model for the log_2_(fold changes (IP/Total)) for eIF4E built from the log_2_(fold changes (IP/Total)) of the four other proteins. This produces a good model with R^2^ = 0.75. The β coefficients for the eIF4E binding partners equated to eIF4E’s mRNA binding profile from this model are displayed in Figure 7C. Each protein makes a highly significant contribution to the model, with *P*-values estimated to be below 0.002 in all cases. The β coefficients for eIF4G1 and eIF4G2 are large and positive, while the coefficient of Caf20p is low and that of Eap1p is negative. These broadly reflect the pairwise GLM correlation values between eIF4E and the other proteins shown in Figure 3, and the similarities in the heat map in Figure 4. For example, the RNA binding profiles of eIF4G1 and eIF4G2 are very closely related to that of eIF4E, whereas the RNA binding profiles of the 4EBPs, Caf20p and Eap1p, are poorly correlated with eIF4E's. We wish to make clear that the β coefficients from the model are not simply explained by the relative abundance of the closed loop components: taking estimates of relative protein abundance from the PaxDb integrated quantitative proteomic dataset [49], the levels of the four yeast proteins are: eIF4E = 1,011 ppm, eIF4G1 = 444 ppm, eIF4G2 = 118 ppm, Caf20p = 306 ppm, Eap1p = 57 ppm. An analysis such as this is complicated by the high degree of co-linearity when comparing the mRNA binding profiles from eIF4G1 with eIF4G2 or those of Caf20p with Eap1p. This co-linearity may account for the larger coefficient for eIF4G2 relative to eIF4G1, even though protein abundances would suggest eIF4G1 should play a more prominent role. However, such co-linearity cannot account for the relatively low coefficient of Caf20p and the negative coefficient of Eap1p. The low and negative coefficients for Caf20p and Eap1p, respectively, are likely to be indicative of a more complex network of interactions with mRNA or other RNA binding proteins that do not rely on eIF4E and the mRNA cap structure.

By far the most striking observation from the modeling is shown in the plot of the jackknife residuals versus the fitted values (Figure 7D). This is a common diagnostic plot in linear regression, where the standardized residuals for individual transcripts should center on a mean of 0 for a well-described model. Therefore, as can be seen in Figure 7D, almost all of the data fit this model well. However, the two mRNAs that lie outside this model to the greatest extent are those encoding the 4E-BPs, Caf20p and Eap1p. This observation strongly suggests that within the confines of the closed loop complex, the translation initiation of the *CAF20* and *EAP1* mRNAs lies outside this model and is regulated in a different manner.

### Caf20p interacts preferentially with and regulates its own transcript

**Figure 8:**
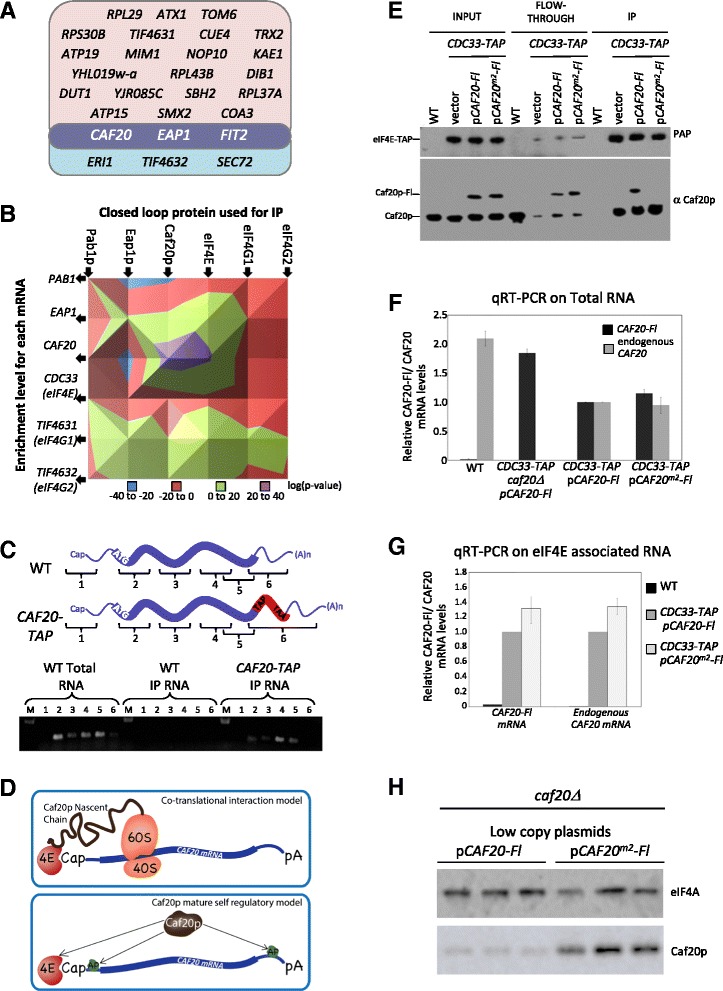
**Caf20p self-regulates its own transcript. (A)** Transcripts overrepresented in the eIF4E pull-downs relative to either eIF4G1 (light blue) or eIF4G2 (pink). The data are taken from the GLM model presented in Figure 3B. **(B)** A three-dimensional surface plot of *P*-values detailing the level of significance for the enrichment of the individual closed loop/eIF4E-BP transcripts across the six RIP-seq experiments. **(C)** A semi-quantitative RT-PCR validation of Caf20p protein’s association with its own transcript using primers designed to the regions 1 to 6 depicted in the figure (detailed in Additional file 6). The level from these regions of the *CAF20* transcript was determined in TAP affinity purified samples from the *CAF20-TAP* strains relative to wild-type (WT) strains. **(D)** A diagram depicting two possible models by which Caf20p could interact with its own transcript to regulate protein production. **(E)** TAP affinity purification and western blot analysis from eIF4E-TAP tagged strains, investigating the association of eIF4E with both endogenous Caf20p protein and Flag-tagged wild-type Caf20p or Flag-tagged Caf20^m2^p (which has had the eIF4E binding region mutated). **(F)** Validation of the specificity of RT-PCR primers using total RNA from the strains depicted under the bar chart for either endogenous *CAF20* transcripts or Flag-tagged *CAF20* transcripts (Additional file 6). Error bars are ± standard error from three replicate experiments. **(G)** qRT-PCR for the endogenous and Flag-tagged *CAF20* transcripts from an eIF4E-TAP affinity purification using the primers validated above. The *CAF20* or *CAF20-fl* transcripts are quantified in the IP samples relative to total RNA for the strains listed. Error bars are ± standard error from three replicate experiments. **(H)** Western blot analysis using extracts from *caf20* deletion strains transformed with either centromeric (low copy) plasmids bearing either wild-type *CAF20-fl* gene or the m2 mutant of *CAF20-fl*. Three different single transformants are analyzed for each strain and the blots are probed with anti-Flag antibodies to detect Caf20-fl relative to control anti-eIF4A antibodies.

The direct statistical pairwise comparison between protein pull-downs (Figure 3) also points to atypical behavior from the *CAF20* and *EAP1* mRNAs. The mRNA binding profiles of eIF4E and eIF4G1 are highly correlated with only a handful of transcripts preferentially enriched in the eIF4E pull-down (Figure 3B). Remarkably, however, among the six statistically enriched transcripts are *CAF20*, *EAP1* and *TIF4632* mRNAs, which encode the 4E-BPs Caf20p and Eap1p, and eIF4G2, respectively (Figure 8A). Similarly, when the eIF4E and eIF4G2 pull-downs were compared, we observed 24 transcripts over-represented in the eIF4E pull-down relative to the eIF4G2 pull-down (Figure 3B). Again mRNAs for *CAF20* and *EAP1*, and, this time *TIF4631*, encoding eIF4G1, were preferentially associated with eIF4E (Figure 8A). These data show that the *CAF20*/*EAP1* mRNAs are enriched with eIF4E but not with eIF4G1.

To further explore the possibility that protein components of the closed loop system are involved in the regulation of mRNAs encoding other components of the system, a statistical analysis based on the significance of any enrichment for each of the transcripts encoding the six immunopurified proteins across the RIP-seq experiments was undertaken. These data are presented as a three-dimensional surface plot in Figure 8B, where peaks (purple) denote significance of transcript enrichment in specific IPs, whereas troughs (blue) represent the significance of any under-representation. By far the most striking relationships on this plot are associated with Caf20p and eIF4E binding of the *CAF20* transcript (Figure 8B). Indeed, *CAF20* is by far the most over-represented transcript in the Caf20p immunoprecipitation with respect to total mRNA from the EdgeR GLM analysis, with a corrected FDR <10^-30^.

On the basis of the results above, we theorized that the interaction of Caf20p and eIF4E with the *CAF20* transcript may represent an autoregulatory mechanism (Figure 8D). Intriguingly, in this regard, previous studies have noted a difficulty in the genetic over-expression of Caf20p: expression of all other components of the closed loop complex from high copy plasmids in yeast leads to a two- to five-fold increase in the level of protein, whereas high copy plasmids bearing the *CAF20* gene generate no such overexpression. However, such a plasmid does generate wild-type levels of Caf20p in a *caf20Δ* strain [52]. In order to directly validate the observation that Caf20p interacts with its own transcript, TAP affinity purifications were performed in wild-type and Caf20p-TAP strains and the associated RNAs were fragmented by RNase III treatment. A semi-quantitative RT-PCR assay was performed on these samples to assess the efficiency of enrichment of six different regions of the *CAF20* transcript (Figure 8C). RT-PCR products were identified from across the *CAF20* mRNA in the immunoprecipitated samples, whereas no products were found in samples from strains bearing untagged *CAF20*. Particular enrichment was observed for primer pairs covering the 3’ end of the open reading frame (Figure 8C). Thus, by use of an independent assay, these data confirm that Caf20p can interact with the *CAF20* mRNA.

Although the data above highlight the possibility that Caf20p autoregulates translation of its own transcript, it is also possible that nascent partially translated Caf20p interacts via its amino-terminal eIF4E binding domain with eIF4E bound to the *CAF20* mRNA 5’ cap (Figure 8D). This could explain the enrichment of the *CAF20* mRNA with eIF4E and Caf20p. Such co-translational interactions of protein-protein complexes have been described previously, and can explain the co-enrichment of mRNAs encoding a specific subunit with other protein subunits of the same complex [53].

To explore this possibility further, we tested whether the enrichment of *CAF20* mRNA with eIF4E is dependent on Caf20p protein association with eIF4E. We used a strategy where Flag-tagged Caf20p or Flag-tagged Caf20^m2^p (where the region of Caf20p which interacts with eIF4E has been disrupted via two missense mutations) were expressed from plasmids in an eIF4E-TAP strain already harboring the wild-type endogenous genomic *CAF20* allele. This places the Flag-tagged mRNA in competition with the endogenous *CAF20* mRNA and allows the impact of the eIF4E binding mutation on this competition to be analyzed. As a control for the system, western blotting of TAP affinity purified eIF4E captured both the endogenous Caf20p and Flag-tagged Caf20p (Figure 8E), whereas when the Flag-Caf20^m2^p mutant protein was placed in competition with endogenous Caf20p, eIF4E only interacted with the endogenous protein (Figure 8E), confirming that the m2 mutations disrupt eIF4E binding [52].

qRT-PCR was used to distinguish the endogenous *CAF20* mRNA from the Flag-tagged *CAF20* mRNA. Control qRT-PCR reactions from total RNA samples demonstrated primer pair specificity (Figure 8F). In a wild-type strain only the endogenous *CAF20* mRNA was detected, while only the tagged form of the mRNA was identified in a strain bearing only the Flag-tagged *CAF20* gene (*caf20Δ Flag-CAF20*; Figure 8F). Finally, in strains bearing both the endogenous *CAF20* and Flag-tagged *CAF20* genes, both mRNAs were detected (Figure 8F). We therefore used this system to measure the level of both the Flag-tagged and endogenous *CAF20* mRNAs with immunoprecipitated eIF4E, and both mRNAs were detected in eIF4E immunoprecipitations. Critically, the Flag-tagged *CAF20* mRNA associated with eIF4E irrespective of whether the protein product of this mRNA could interact with eIF4E (compare the results for strains bearing p*CAF20-Fl* versus p*CAF20*^*m2*^*-Fl*; Figure 8G). Therefore, the interaction of eIF4E with the *CAF20* mRNA does not rely on the capacity of the Caf20p protein to interact with eIF4E. From these data, we posit that it is highly unlikely that the prime reason for the selective enrichment of *CAF20* mRNA with either eIF4E or the Caf20p protein is the 'cotranslational interaction' of nascent Caf20p via its amino-terminal eIF4E binding domain. Instead, we favor the model where mature Caf20p protein selectively enriches the *CAF20* mRNA presumably via Caf20p interactions with other RNA binding proteins as well as its interaction with eIF4E (Figure 8D). Indeed, Caf20p has been previously shown to have interactions with both the Puf4p and Puf5p RNA binding proteins [40].

A prediction of the Caf20p self-regulatory model is that in strains where the eIF4E binding mutant (Caf20^m2^) is the sole source of Caf20p, the self-regulation will be short-circuited and Caf20p will accumulate to higher levels than in strains bearing wild-type Caf20p. Indeed, in Figure 8H this prediction is found to be correct: in *caf20Δ* mutants bearing the p*CAF20*^*m2*^*-Fl* plasmid, Caf20p accumulates to 7.05 (±1.25)-fold higher levels than in the mutant bearing the wild-type plasmid. Taken collectively, these data highlight an autoregulatory circuit controlling Caf20p expression in a negative feedback loop, where a self-limiting brake is applied to modulate the expression of a general cellular translational repressor.

## Conclusions

This study represents a systematic analysis of the mRNA binding profiles for the components involved in the formation of the closed loop complex and its regulation. Several striking features are observed. We identify two mRNA populations that encode highly abundant, heavily translated proteins: as predicted by the closed loop model, one set is enriched in immunoprecipitations of the closed loop components (group II), whereas unexpectedly the other set is apparently under-represented for all components except Pab1p (group I). Intriguingly, the mRNAs that are enriched with the closed loop encode ribosomal and ribosomal biosynthetic proteins, highlighting similarities with the TOP mRNAs in mammalian systems. Those mRNAs that are under-enriched for closed loop components indicate that alternatives to the closed loop complex likely exist to allow the direction of ribosomes to these mRNAs, though we wish to emphasize these are still abundant, highly translated RNAs. A variety of studies have suggested alternatives to the widely accepted eIF4E-eIF4G-PABP mode of translation initiation in yeast [54–56]. These include the use of internal ribosome entry sites, alternative closed loop complexes and a discrete function of PABP. A possibility from our data, which is consistent with these other studies, is that Pab1p would be somehow involved in such a mechanism.

Other classes of mRNA identified are those that are heavily enriched with the 4E-BPs, where it seems likely that an equilibrium exists between closed loop components and the 4E-BPs. Our data highlight how the 4E-BP binding properties appear to be antagonistic with eIF4E and eIF4G proteins, and particularly Pab1p, consistent with their general role as translation repressors. Notably, however, as part of our analysis of these data, we uncovered the potential that the 4E-BPs are self-regulated at the level of translation initiation. Although the self-regulation in terms of levels of Caf20p protein produced relies upon interactions with eIF4E, the interaction of Caf20p with the *CAF20* mRNA does not and one possibility is that other RNA binding protein interactions are more important for the targeting to the *CAF20* mRNA.

## Materials and methods

### Strains and growth conditions

Strains used in this study are listed in Additional file 6. TAP-tagged His^+^ strains in the BY4741 background where obtained from Thermo Scientific Open Biosystems (Waltham, MA, USA). Strains were generally grown at 30°C in synthetic complete dextrose media lacking Histidine (SCD-His) [57]. An untagged *HIS3* BY4741 control strain was generated as a control for all experiments by restoring the *HIS3* endogenous gene via chromosomal integration of a *Xho*I-*Bam*HI fragment from pUN90 [58] at the genomic *HIS3* locus. The *CDC33-TAP::HIS3* strain from Open Biosystems was found to increase eIF4E (Cdc33p) expression levels, and concurrently decrease Caf20p expression levels, when compared with the BY4741 wild-type strain (data not shown). A new *CDC33-TAP* strain, yMK2198, was generated where the selectable marker was removed after cassette integration. To achieve this, a pUC57-based plasmid, BMK722, bearing TAP upstream of a *Loxp-URA3-LoxP* cassette, was synthesized and used in a standard PCR-based S2/S3 endogenous gene-tagging protocol [59]. The *URA3* marker was removed by site-specific recombination using the standard Loxp Cre-recombinase system [60]. In this new strain, expression levels of eIF4E and Caf20p were restored to wild type (data not shown). Strains yMK2201/2/3 were generated by transformation of yMK2198 with control vector, or plasmids expressing Caf20p-FLAG(Fl) or a 4E-binding mutant form of Caf20p-Fl.

### TAP-affinity purification

Yeast cultures were grown to an OD_600_ of 0.6, pelleted and snap frozen in liquid nitrogen. Samples were ground in Buffer A (20 mM Tris-HCl (pH 8), 140 mM NaCl, 1 mM MgCl_2_, 0.5% NP40, 0.5 mM DTT, 1 mM PMSF, EDTA free Protease Inhibitor cocktail tablet (Roche Diagnostics, Indianaplois, IN, USA), 100 μM NaV_3_O_4_, 5 mM NaF and 40 units/ml RNasin (Promega, Flitchburg, WI, USA)) with liquid nitrogen in a 6870 Freezer Mill (Spex, Metuchan, NJ, USA), and cleared through two centrifugation steps of 15,000 × *g* at 4°C. Five percent of the lysate was reserved for isolation of total RNA. Lysates were quantified and varying concentrations of total protein (10 mg CDC33-TAP; 10 mg TIF4631-TAP; 10 mg TIF4632-TAP; 1 mg PAB1-TAP; 10 mg CAF20-TAP; 25 mg EAP1-TAP) loaded onto Tosyl-activated Dynabeads M-280 magnetic beads (Life Technologies, Carlsbad, CA, USA) to ensure maximum depletion of the tagged proteins.

Coupling of rabbit IgG to Tosyl-activated Dynabeads M-280 magnetic beads and TAP affinity purification were performed as previously described [61]. After the final wash, the beads were re-suspended in 270 μl Buffer A. A 20 μl aliquot of the sample was set aside for western blot analysis, and RNA was purified from the remaining 250 μl. Total RNA and IP RNA were isolated via the addition of 750 μl Trizol Reagent (Life Technologies) and 200 μl chloroform to the 250 μl samples. After extraction, the aqueous phase was collected, and precipitated overnight at -20°C with 500 μl isopropanol and 1 μl glycogen (10 mg/ml). The pelleted RNA was washed twice with 1 ml 70% ethanol in diethylpyrocarbonate (DEPC) water and re-suspended in 10 μl DEPC water. RNA was quantified using a Nanodrop 8000 spectrophotometer (Thermo Fisher Scientific).

For the Caf20-TAP semi quantified RT-PCR experiment, the following modifications were made to the above TAP affinity protocol. A formaldehyde cross-linking step was introduced by rapidly chilling the culture with 1% (v/v) formaldehyde for 1 hour in ice-water. Cross-linking was terminated with 0.1 M glycine, and cultures pelleted and processed as described above. In addition, following the final immunoprecipitation wash, two further washes were performed with RNAse III buffer (10 mM Tris, pH8, 10 mM MgCl_2_, 1 mM DTT, 60 mM NaCl, 10U/ml RNasin). Samples were re-suspended in RNAse III buffer containing 40U RNasin, 1U DNAse I (Promega) and 2U RNAse III (New England Biolabs, Ipswich, MA, USA) and incubated for 10 minutes at 37°C. Following digestion, RNase III was deactivated by the addition of 900 μl Buffer A-EDTA (Buffer A with 1 mM EDTA but without MgCl_2_), followed by a 5 minute wash in Buffer A-EDTA at 4°C. Samples were then sequentially washed for 5 minutes at 4°C with Buffer A-500 (Buffer A with 500 mM NaCl), then Buffer A-250LiCl (Buffer A with 250 mM LiCl). A proteinase K digestion step was then introduced by first washing with proteinase K buffer (10 mM Tris, pH8, 200 mM NaCl, 1 mM EDTA, 0.1% SDS, 10 U/ml RNasin) for 5 minutes at 4°C, followed by digestion in this buffer with 100 μg/ml proteinase K for 30 minutes at 42°C. Samples were then heated to 65°C for 60 minutes to reverse crosslinks and the RNA was processed as above.

### Preparation of sequencing libraries

Total RNA samples were normalized to the amount of RNA isolated from the corresponding IP sample. rRNA was then depleted from the RNA samples using the Ribominus™ Eukaryote Kit for RNA-Seq (Life Technologies). Depleted samples were ethanol precipitated, washed twice with 70% ethanol and resuspended in 10 μl DEPC water. rRNA depletion was checked on a 2100 Bioanalyzer (Agilent Technologies, Palo Alto, CA, USA) using a RNA nanochip and the remaining RNA stored at -80°C.

Sequencing libraries were generated using the whole Transcriptome Library Preparation protocol provided with the SOLiD® Total RNA-Seq Kit (Life Technologies). Briefly, rRNA depleted samples were fragmented using RNase III, and subsequently cleaned up using the RiboMinus™ Concentration Modules (Life Technologies). Fragmentation was assessed on a 2100 Bioanalyzer (Agilent Technologies) using the RNA picochip. Fragmented RNAs were reverse transcribed and size selected on a denaturing polyacrylamide gel selecting for 150 to 250 nucleotide cDNA. cDNA was then amplified and barcoded with SOLiD™ RNA Barcoding Kit. Samples were then purified using PureLink™ PCR Micro Kit (Life Technologies) and assessed on a 2100 Bioanalyzer (Agilent Technologies) using the High Sensitivity DNA chip. Samples were deposited on slides, and sequenced using the SOLiD v4 sequencing system (Life Technologies).

### Reverse transcriptase PCR

For RT-PCR experiments, isolated RNA was converted to cDNA using a Protoscript M-MuLV *Taq* RT-PCR kit (New England Biolabs). For confirmation of the transcript groups, primer pairs were designed for four representative RNAs from each group (Additional file 6). For the Caf20p self-regulation experiments, primer pairs were designed either across the *CAF20* gene or to distinguish between the endogenous or *CAF20-FLAG* mRNA (Additional file 6). For semi-quantitative RT-PCR, PCR products were generated using *Taq* 2xMaster Mix (New England Biolabs) with a standard PCR program of 24 cycles. The transcript cluster validation and eIF4E-TAP qRT-PCR were performed using the CFx Connect Real-Time system with iTaq Universal SYBR Green Supermix (BioRad Laboratories, Hercules, CA, USA). Samples were run in triplicate and normalized to the input RNA for each primer pair used.

### Next-generation sequence analysis

Reads were mapped to the *S. cerevisiae* genome (genome assembly EF4 downloaded from ENSEMBL) using Bowtie version 1 [62]; sequences were then assigned to genomic features using HTseq-count (mapping against the corresponding EF4 GTF file), excluding those mapping to non-coding features. Full mapping statistics are provided in Additional file 2. The raw counts were then processed by EdgeR [44] to calculate statistical significant enrichments of transcripts in the protein IPs relative to TAP-tag whole extracts, using the GLM functionality with a paired statistical design [44]. This generated gene lists with significant over- or under-enrichment in the IPs at a FDR < 0.05. In addition, the GLM functionality was used to measure protein specific variance between experiments, comparing each IP to each other in a pairwise fashion, through the use of an interaction model [45] and again assigning significance at an FDR < 0.05. This identifies mRNAs with differential enrichment between paired IPs.

Fold changes are presented as log_2_ ratios of counts per million (transcripts with fewer than 20 reads in each of the pertinent total extract samples were excluded from the plots). No further normalization was performed, thus allowing a direct raw comparison between all the datasets. A consequence of this is that the fold changes are subject to 'real estate' effects. That is, fold changes of high abundance transcripts can shift the mean fold change from zero (as is evident in Figure 3). However, these effects are accounted for in the edgeR protocol for determining statistical enrichment.

The IP-enrichment profiles for the combined set of 3,173 yeast transcripts determined to be statistically over- or under-enriched in at least one of the six IPs according to EdgeR’s GLM model at FDR <0.01 and with 20 raw counts in all total RNA samples were subject to hierarchical clustering using Cluster [62]. The uncentered Pearson correlation coefficient was used as a similarity metric with the average linking method. Four natural major clusters of differentially enriched genes containing 2,767 transcripts were defined manually from inspection of the attendant heatmap and dendrogram.

The complete IP-enrichment profiles for all yeast transcripts were also used to model the profile eIF4E association, using the *lm* Multiple Linear Regression functionality within R. The four binding profiles were represented as log_2_ fold change (IP/Total) values, calculated from normalized counts restricted to genes with more than 20 counts in all total runs. Model fitting generated coefficients (β values in Equation 1.1), representing strength of interaction of each profile in the stoichiometric model of eIF4E interaction, reasoning the eIF4E profile itself is a proxy for a rate-limiting initiation step in translational control.

Sequencing data are publicly available from ArrayExpress, E-MTAB-2464.

### Western blot analysis

The 20 μl IP aliquots were mixed with 20 μl 2 × SDS loading dye and heated to 95°C for 10 minutes to dissociate protein complexes from the IgG Tosyl-activated Dynabeads M-280 magnetic beads. IP samples were resolved by SDS-PAGE, electroblotted onto nitrocellulose membrane and probed using the relevant primary antibody. TAP-tagged proteins were detected using an horseradish peroxidase (HRP)-conjugated primary antibody to Protein A (Abcam, Cambridge, MA, USA). All other primary antibodies were detected with HRP-conjugated rabbit secondary antibody, with the exception of Pab1p, which was detected using HRP-conjugated mouse secondary antibody.

### Motif analysis

Motif discovery was performed using REFINE, searching in the 5’ and 3’ UTRs and coding regions of enriched transcripts, defined in the EF4 GTF genome annotation from Ensembl, using the same parameters as defined previously [64].

### Gene Ontology analysis

GO-Slim terms were downloaded from SGD [65]. Significant co-association with enriched transcripts was determined using the hypergeometric test, corrected for multiple testing using an established correction [66]. The significance of statistically enriched terms (given a 0.01 FDR cutoff, in at least one of the datasets) was visualized using TreeView [63].

## Additional files

Additional file 1:
**Experiments addressing the validity of using TAP-tagged strains.**
Additional file 2:
**Sequencing statistics and gene lists corresponding to the transcripts enriched in the individual IP experiments. Sheet 1.** Summary of mapping statistics. **Sheet 2.** Mapping statistics by library. **Sheet 3.** Mapping quality control for a subset of the data. **Sheet 4.** Counts by gene. **Sheet 5.** Full IP/Total ratios for Eap1p. **Sheet 6.** Full IP/Total ratios for Caf20p. **Sheet 7.** Full IP/Total ratios for eIF4G2. **Sheet 8.** Full IP/Total ratios for eIF4G1. **Sheet 9.** Full IP/total ratios for eIF4E. **Sheet 10.** Full IP/Total ratios for Pabl1p. **Sheet 11.** Total normalized counts for various gene cluster groups. Additional file 3:
**Comparison of the Caf20p and Eap1p RIP-seq datasets with translational profiling data from the** ***caf20Δ*** **strain.**
Additional file 4:
**A list of transcripts at variance to the interaction model (derived from the Generalised Linear Model (GLM) function of EdgeR) in pairwise comparisons of the IP enrichment profiles. Sheet 1.** A comparison of IPs. **Sheet 2.** Gene names. Additional file 5:
**The complete lists of gene clusters.**
Additional file 6:
**Lists of genes and description for each group identified in hierarchical clustering.**

## Abbreviations

4E-BP: eIF4E-binding protein
DEPC: diethylpyrocarbonate
eIF: eukaryotic translation initiation factor
FDR: false discovery rate
GLM: Generalised Linear Model
HRP: horseradish peroxidase
IP: immunopurification
PABP: poly(A) binding protein
PCR: polymerase chain reaction
qRT-PCR: quantitative reverse transcriptase PCR
TAP: tandem affinity purification
TOP: terminal oligopyrimidine
UTR: untranslated region
