# Supplementary figures and images for: Global mRNA selection mechanisms for translation initiation
Source: Genome Biol. 2015 Jan 5;16(1):10. doi: 10.1186/s13059-014-0559-z (PMC4302535; doi:10.1186/s13059-014-0559-z)

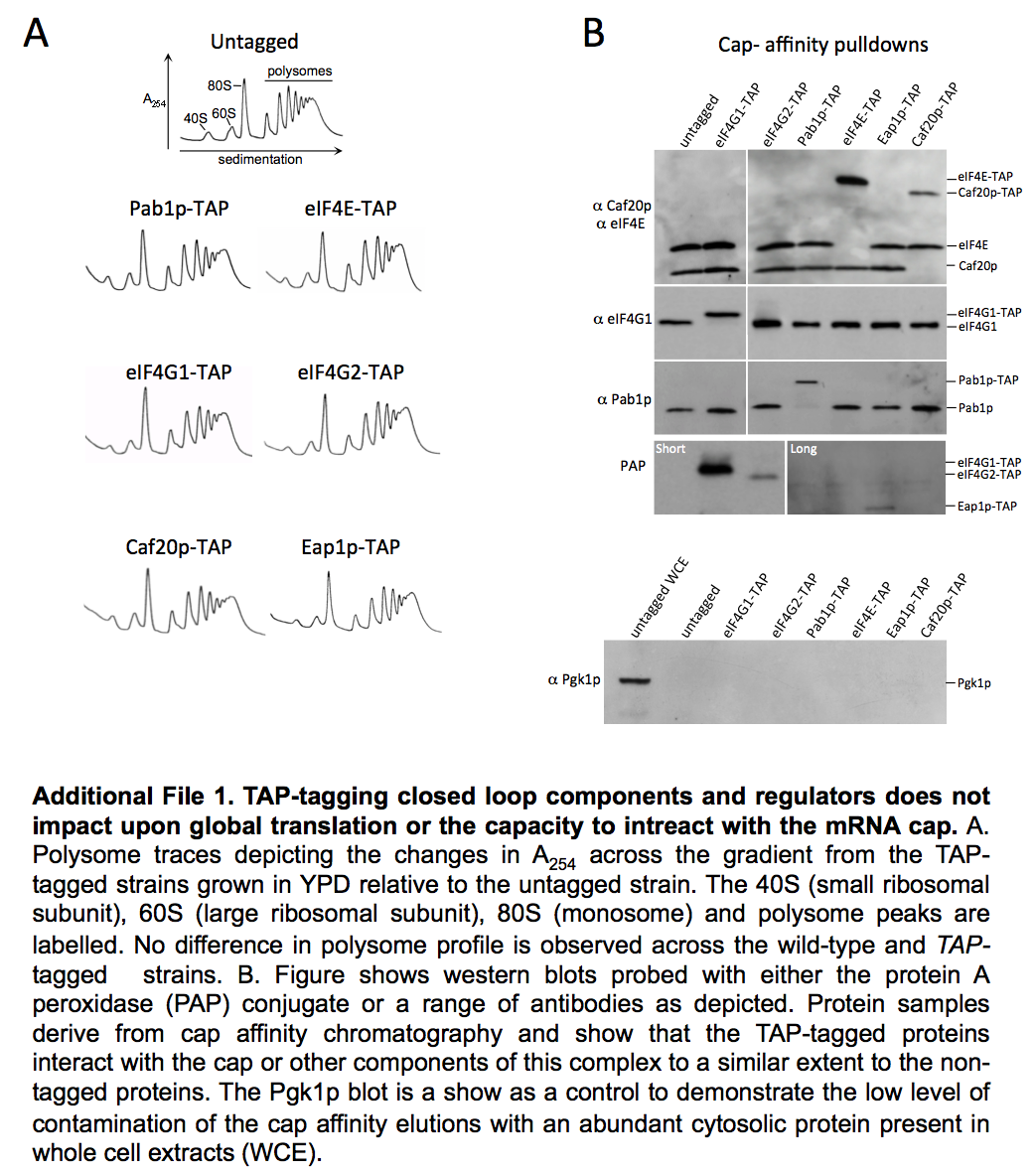

Supplement: Additional file 1: — Experiments addressing the validity of using TAP-tagged strains. [file 13059_2014_559_MOESM1_ESM.png]

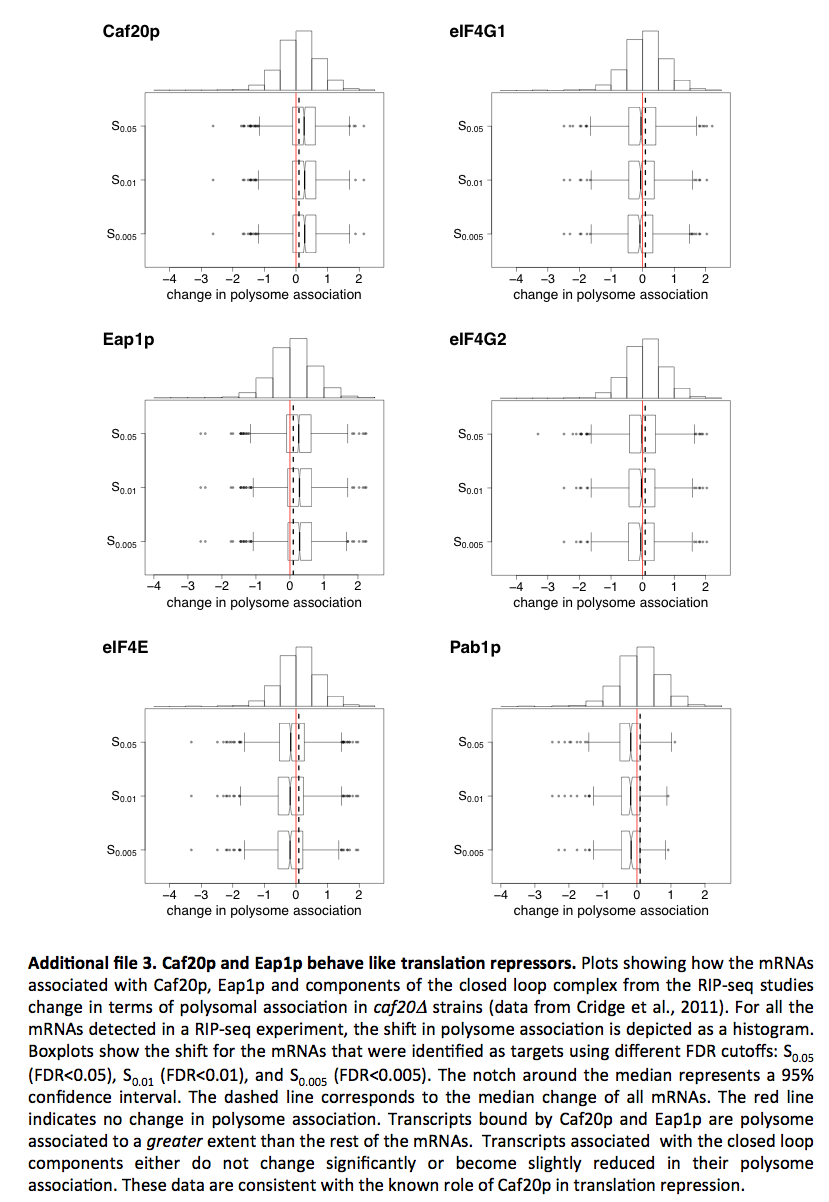

Supplement: Additional file 3: — Comparison of the Caf20p and Eap1p RIP-seq datasets with translational profiling data from the caf20Δ strain. [file 13059_2014_559_MOESM3_ESM.png]
