# Supplementary material for: Global mRNA selection mechanisms for translation initiation
Source: Genome Biol. 2015 Jan 5;16(1):10. doi: 10.1186/s13059-014-0559-z (PMC4302535; doi:10.1186/s13059-014-0559-z)
Supplement: Additional file 6: — Lists of genes and description for each group identified in hierarchical clustering. [file 13059_2014_559_MOESM6_ESM.docx]

| **Yeast strains table** | | |  |
| --- | --- | --- | --- |
| Strain | Genotype | Source | |
| yMK2197  yMK2198  yMK2084  yMK2085  yMK2088  yMK2199  yMK2200  yMK1781  yMK2201  yMK2202  yMK2203 | *MATa HIS3 leu2Δ0 met15Δ0 ura3Δ0*  *MATa HIS3 leu2Δ0 met15Δ0 ura3Δ0 CDC33-TAP*  *MATa his3Δ1 leu2Δ0 met15Δ0 ura3Δ0 TIF4631-TAP::HIS3*  *MATa his3Δ1 leu2Δ0 met15Δ0 ura3Δ0 TIF4632-TAP::HIS3*  *MATa his3Δ1 leu2Δ0 met15Δ0 ura3Δ0 PAB1-TAP::HIS3*  *MATa his3Δ1 leu2Δ0 met15Δ0 ura3Δ0 CAF20-TAP::HIS3*  *MATa his3Δ1 leu2Δ0 met15Δ0 ura3Δ0 EAP1-TAP::HIS3*  *MATa HIS3 leu2Δ0 met15Δ0 ura3Δ0 caf20::G418 p[CAF20-Fl URA3 2μ]*  *MATa HIS3 leu2Δ0 met15Δ0 ura3Δ0 CDC33-TAP p[URA3 2μ]*  *MATa HIS3 leu2Δ0 met15Δ0 ura3Δ0 CDC33-TAP p[CAF20-Fl::URA3 2μ]*  *MATa HIS3 leu2Δ0 met15Δ0 ura3Δ0 CDC33-TAP p[CAF20Δ4E-Fl::URA3 2μ]* | This study  This study  Open Biosystems  Open Biosystems  Open Biosystems  Open Biosystems  Open Biosystems  This study  This study  This study  This study | |

| **Oligonucleotide table:** Oligonucleotides used in this study | | |
| --- | --- | --- |
| Oligonucleotide | Sequence (5’ to 3’) | Use |
| TEF1 F  TEF1 R  ENO2 F  ENO2 R  PDC1 F  PDC1 R  ACT1 F  ACT1 R  TOM5 F  TOM5 R  ATP15 F  ATP15 R  RPS20 F  RPS20 R  RPL38 F  RPL38 R  DIG1 F  DIG1 R  SKS1 F  SKS1 R  ALY1 F  ALY1 R  YIR016W F  YIR016W R  PRO1 F  PRO1 R  GCN20 F  GCN20 R  VTC4 F  VTC4 R  NIC96 F  NIC96 R  Caf20 1 F  Caf20 1 R  Caf20 2 F  Caf20 2 R  Caf20 3 F  Caf20 3 R  Caf20 4 F  Caf20 4 R  Caf20 5 F  Caf20 5 R | CAACGCTACCGTCATTGTTTT  GATGGAACGAACTTGACCAAA  ATCCGAAAGATTGGCTAAGTTG  CTTGTCACCGTGGTGGAAG  ATTGCTGACGCCGCTAAGG  GGTTCCACATCCATTCTTGCTTC  TGACTGAAGCTCCAATGAACC  TACTCTTCCGGTAGAACTACTG  TTTCACCAAATGCCAGATCA  CAGGAAGTCTCCGAAGAGGA  GCCTGGAGGAAAGCTGGTAT  AGAAGCTGCAGTGCCATTTT  GGTCAAGAAGGGTCCAGTCA  CAACTTCGACATCCACACCA  GACGTTAAGACCGCCACTGT  AAGTTGGTGGCAAAGATTGG  ACTTGGGAAAGGCCAGTTCT  AGGGTGTCATTATGGGCTTG  AGGGAGATTGCCTTCCAACT  GATCTAATGCGGAGCAAAGC  AACGGTCCTAAACTGCCTCA  CAATGGTGGGCTTTGATTTT  GTTCCGGCAGAACAAGAAAA  GAGCGTACTGAGCGTCTTCC  TTGCCAGCAGGTGTTATTGA  AGCCCAATTCCTCTTCGATT  ACCGGTGATACGTCCAAGAG  CTCTTGGCGACTTTCTTTGC  TTGAGCCAGTCCCAAAATTC  CGTCATCCTCATCATCCTCA  TGTCGAGTTATGCCATGAGG  GTCTTCGTCTGCCCTTCTTG  GGCGTAAAGCATATTCTACCG  ACTTGATCATGTCGTGAAATTTA  AGCTTTTTCAACTGAAGCCAA  GTGTTGCAATTGCTTAACTTT  ATGGGCTACCATTGCCCAAGA  GGCTTGTCCGCAACAATATCT  TCTTGGTTTCAACGCATTTGC  TAGTACACGTACAGCTGCCTG  GTCATTCGCTCATGCTTTCTT  AGGTTACTTTCCAGATTGAAA | CLUSTER VALIDATION  CLUSTER VALIDATION  CLUSTER VALIDATION  CLUSTER VALIDATION  CLUSTER VALIDATION  CLUSTER VALIDATION  CLUSTER VALIDATION  CLUSTER VALIDATION  CLUSTER VALIDATION  CLUSTER VALIDATION  CLUSTER VALIDATION  CLUSTER VALIDATION  CLUSTER VALIDATION  CLUSTER VALIDATION  CLUSTER VALIDATION  CLUSTER VALIDATION  CLUSTER VALIDATION  CLUSTER VALIDATION  CLUSTER VALIDATION  CLUSTER VALIDATION  CLUSTER VALIDATION  CLUSTER VALIDATION  CLUSTER VALIDATION  CLUSTER VALIDATION  CLUSTER VALIDATION  CLUSTER VALIDATION  CLUSTER VALIDATION  CLUSTER VALIDATION  CLUSTER VALIDATION  CLUSTER VALIDATION  CLUSTER VALIDATION  CLUSTER VALIDATION  Caf20 CLIP  Caf20 CLIP  Caf20 CLIP  Caf20 CLIP  Caf20 CLIP  Caf20 CLIP  Caf20 CLIP  Caf20 CLIP  Caf20 CLIP  Caf20 CLIP |

**Additional File 6. Yeast strains and oligonucleotides used in this study.**
